# Seronegative celiac disease: where is the specific setting? 

**Published:** 2015

**Authors:** Enzo Ierardi, Giuseppe Losurdo, Domenico Piscitelli, Floriana Giorgio, Claudia Sorrentino, Mariabeatrice Principi, Lucia Montenegro, Annacinzia Amoruso, Alfredo Di Leo

**Affiliations:** *Section of Gastroenterology, Department of Emergency and Organ Transplantation, University of Bari, Italy *

**Keywords:** Seronegative celiac disease, Tissue transglutaminase, Imunoglobulins, Serology, Diagnosis

## Abstract

The diagnosis of Celiac Disease (CD) relies on the concordance of pathological, serological, genetic and clinical features. For this reason, the diagnosis of CD is often a challenge. Seronegative celiac disease (SNCD) is defined by the negativity of anti-tissue transglutaminase antibodies in the presence of a positive histology on duodenal biopsy samples, i.e. inflammatory infiltrate of intra-epithelial lymphocytes (IELs > 25/100 enterocytes), mild villous atrophy and uneven brush border associated to human leukocyte antigen (HLA) haplotype DQ2 and/or DQ8.

SNCD is characterized by mucosal deposits of tissue transglutaminase (tTG)/anti-tTG immuno-complexes. These may counteract the passage of anti-tTG into the bloodstream, thus explaining seronegativity. Another reason for seronegativity may be found in an incomplete maturation of plasma cells with a consequent failure of antibodies production. This condition often characterizes immunoglobulin deficiencies, and, indeed, SNCD is common in subjects with immunoglobulin deficiencies.

The management of SNCD still remains debated. The treatment option for SNCD may be represented by gluten free diet (GFD), but the usefulness and appropriateness of prescribing GFD are controversial. Some evidences support its use only in SNCD subjects showing CD clear clinical picture and compatible HLA status. The choice of GFD administration could be linked to an investigation able to diagnose SNCD in no doubt even if a reliable test is not currently available. On these bases, a test helping the diagnosis of SNCD is justifiable and desirable.

## Definition of seronegative celiac disease

Celiac disease (CD) is an autoimmune enteropathy characterized by villous atrophy and lymphocytic inflammation of the epithelial layer covering the mucosa (1). In detail, CD is characterized by CD3-positive T-lymphocyte inflammatory infiltration, i.e. intraepithelial lymphocytes (IELs) (2). Marsh and Oberhuber, who elaborated a three-degree classification of duodenal mucosal pattern in CD, have described a grading of the epithelial damage in CD. In the grade 1, a lymphocytic infiltrate without villous atrophy is present, and more than 25 IELs per 100 enterocytes are observed. The elongation of the cripts, with a cript/villum ratio of 1:2 or 1:3, characterizes the grade 2 while the normal ratio is conventionally 1:5. Finally, in the grade 3 the villous atrophy is the dominant feature (3).

However, the diagnosis of CD is not only based on pathological findings. The serological assessment of autoantibodies associated to this disease is essential to achieve a final diagnosis (4). The autoantibodies that are commonly related to CD are IgA anti-tissue transglutaminase 2 (IgA anti-tTG) and the anti-endomysium (EMA). Anti-gliadin antibodies (AGA) are considered as less relevant for their low sensitivity and specificity and have been replaced by anti-deamidated gliadin peptide (DGP), which has a better performance, as suggested by current guidelines (5). A profile of sensitivity and specificity of such antibodies is displayed in table 1.

**Table 1:** Sensitivity and specificity of main autoantibodies used in clinical practice for the serological diagnosis of CD (adapted from Armstrong et al. ref. 4)

Test	Sensitivity %	Specificity %
IgA-AGA	46-87	70-98
IgG-AGA	42-93	84-97
IgA-DGP	75-78	95-100
IgG-DGP	65-71	95-98
EMA	74-100	99-100
IgA-tTG	81-100	97-99
IgG-tTG	27-100	77-95

AGA: anti-gliadin; DGP: anti deamidated gliadin peptide; tTG: anti tissue transglutaminase; EMA: anti-endomysium

Human leukocyte antigen (HLA) haplotype DQ2 and/or DQ8 are associated to most cases of CD, (4) which is characterized by known clinical symptoms (1).

On these bases, the diagnosis of CD relies on the concordance of pathological, serological, genetic and clinical features. For this reason, the diagnosis of CD is often a challenge (6). The possibility that not all tests may confirm the suspicion is frequent. Therefore, novel nosological entities, such as seronegative celiac disease (SNCD), have been proposed in the spectrum of gluten-related disorders in recent years (7).

SNCD is defined by the negativity of anti-tissue transglutaminase antibodies in the presence of a positive histology on duodenal biopsy samples, i.e. inflammatory infiltrate of intra-epithelial lymphocytes (IELs >25/100 enterocytes), mild villous atrophy and uneven brush border associated to human leukocyte antigen (HLA) haplotype DQ2 and/or DQ8 (4).

## Seronegativity in CD – a narrative overview

In literature, the first study analyzing the problem of SNCD dates back to 2004 (8). This paper aimed to consider the sensitivity and specificity of serological tests, and in particular EMA, in conditions of villous atrophy (Marsh 3) or in the absence of atrophy (Marsh 1 and 2). Results showed that EMA were positive in 77% of atrophic and only in 33% of non-atrophic lesions. The study also analyzed IgA anti-tTG. Although the subjects undergoing this test were only 14, IgA anti-tTG was positive in all the patients with atrophy and absent in those with partial atrophy. Despite a low reliability due to the poor sample size, these data revealed a parallel pattern of both autoantibodies, which target the same antigen, the tTG (9).

The low positivity rate of anti-tTG in non-atrophic enteropathy may be strictly linked to SNCD, since this entity is not always characterized by complete villous atrophy. Several authors have shown that the rate of positivity of anti-tTG correlates directly with the degree of atrophy. Therefore, atrophic lesions are related to positive serology (10), and in this subset of patients most of SNCD cases are scanty represented. Other studies investigating this aspect are shown in table 2 (8, 10-16).

## The pathogenesis of seronegative celiac disease

About the possible pathogenesis of SNCD, it has been speculated that this condition may be due to the lack of passage of autoantibodies produced in the intestine into the circulation. The production of antibodies in individuals with CD occurs in the intestinal mucosa, as evidenced by the presence of immune-complexes detectable by immunofluorescence. Auto-antibodies, successively, cross the mucosa and enter into blood vessels (17). 

**Table 2:** Prevalence of anti-tTG and EMA in cases of non-atrophic CD.

Reference	anti-tTG positivity	EMA positivity
Abrams JA et al, Dig Dis Sci, 2004 (8)	0%	33%
Tursi A et al, J Clin Gastroenterol, 2003 (10)	7.69%	Not tested
Tursi A et al, J Clin Gastroenterol, 2003 (11)	17,1%	8.6%
Dickey W et al, Scand J Gastroenterol, 2000 (12)	Not tested	79%
Tursi A et al, Am J Gastroenterol, 2001 (13)	Not tested	33%
Rostami K et al, Am J Gastroenterol, 1999 (14)	Not tested	31%
Kurppa K et al, J Pediatr, 2010 (15)	88%	Not tested
Salmi TT et al, Gut, 2006 (16)	28.6%	87.6% (cumulative value enclosing atrophic CD)

However, in SNCD the antibodies would be confined in the lamina propria and could not pass into the bloodstream. In this regard, it has been shown that in SNCD IgA anti-tTG has a great affinity for their antigen. Therefore, they strongly bind to tTG2, thus inducing immunocomplex deposits unable to pass into circulation. The deep antigen-antibody connection could explain the negativity of serological tests (16).

Some evidences confirm this hypothesis. Tosco et al. (18) have found, by immunofluorescence, deposits of tTG complexed with IgA anti-tTG in a pediatric population affected by seronegative non- atrophic CD at Marsh 1 stage. These antigen-antibody deposits have been shown to be specific in both evident/seropositive and moderate/seronegative celiac enteropathy. Indeed, they are present in the 90% of cases, with a better efficiency compared to serological tests (19, 20). A randomized study performed on 41 subjects with an increase of IELs at duodenal histology showed that 11 of them were responsive to GFD. Moreover, deposits of immunocomplexes, detected by immunohistochemistry, were highlighted in 10 of the 11 above-mentioned patients (21).

Another possible explanation for seronegativity in CD could be linked to an immaturity of immune system. Indeed, CD is frequently associated to a dysregulation of immune system, in particular immunoglobulin deficiencies such as selective IgA deficiency (sIgAD) or common variable immunodeficiency (CVID) (22-24). In these disorders, it may be presumable that a lack of maturation of plasma cells leads to the inefficient production of autoantibodies directed against the tTG, thus explaining the seronegativity. Even in these cases, deposits of tTG may be retrieved in duodenal samples, as underlined by a recent case report in which SNCD was unmasked by measuring the levels of mRNA codifying tTG in the diseased mucosa of a patient with selective IgM deficiency (25). At baseline, levels of mRNA-tTG were higher than controls, but returned to normal values after one year of gluten-free diet (GFD). In this regard, SNCD diagnosis could be a hard task, since immunoglobulin deficiency may show a histological intestinal damage, which is similar to that of CD. However, this picture may not be related to gluten, but due to infectious agents (26). A group of skilled gastroenterologists and pathologists have underlined that in such doubtful cases, only the histological improvement after a GFD may represent a reliable tool for diagnosing CD or SNCD in subjects with immunoglobulin deficiencies (27).

## To treat or not to treat?

**Figure 1:**
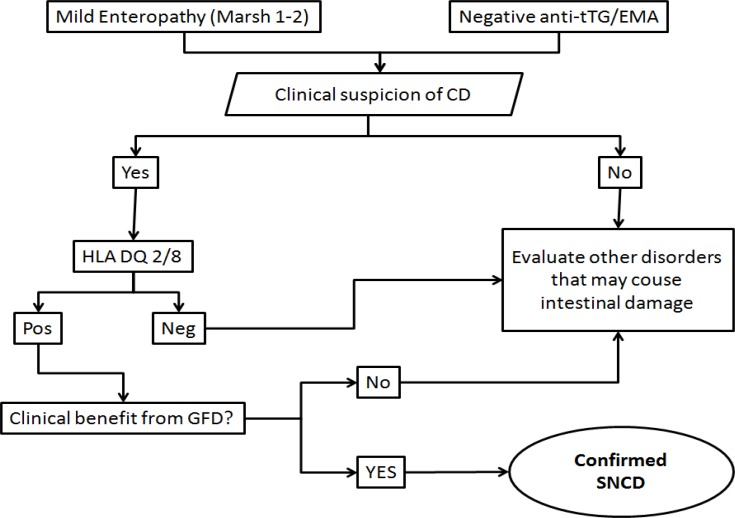
A possible algorithm for the diagnosis of SNCD. Adapted and modified from Leffler et al, ref 28.

The management of SNCD still remains debated. The treatment option for SNCD is represented by the GFD, but its usefulness and appropriateness are controversial. In an editorial of 2013, Leffler et al. (28) have argued that SNCD should not be admitted to GFD, because diet does not improve the quality of life as well as patients do not show metabolic or nutritional deficiencies. However, this statement may be somewhat questionable, taking into account some case reports describing severe nutritional deficiencies in SNCD (29). Finally, the diagnostic pathway of SNCD could recommend the GFD in some conditions, as shown in the diagram reported in Figure 1. In multi-step classification of gluten related disorders according to the entity of adaptive immune system involvement, SNCD could be treated with GFD when clinical and genetic features are strongly evident.

**Figure 2:**
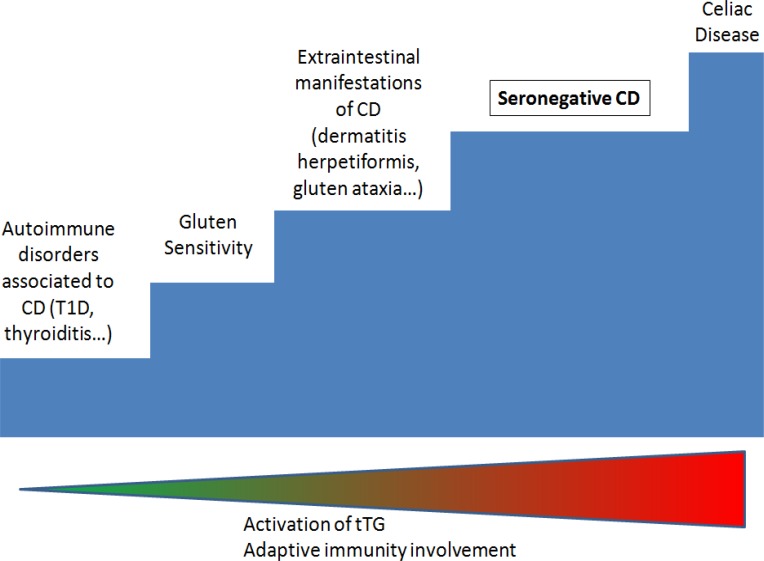
A multi-step classification of gluten related disorders according to the entity of adaptive immune system involvement. T1D: type-1 diabetes. Adapted and modified from Troncone et al. ref 32.

Although rational, this suggestion conflicts with the observation that SNCD is rarely asymptomatic and often underlies an important clinical involvement (30). For example, Zanini et al. (31) showed that mild or seronegative CD experienced weight loss, gastrointestinal and extraintestinal symptoms and other associated autoimmune conditions in the same proportion of patients with atrophic/seropositive CD. This aspect induced some authors to investigate whether a GFD could be of benefit for SNCD. In a study by Tursi et al. (11), SNCD twenty-three patients (7 at Marsh 1 stage and 16 at Marsh 2 stage at baseline) were included in the study protocol and underwent GFD for 8-12 months. After the GFD, 5 out of the 7 cases of Marsh 1 reached the mucosal healing, i.e. a Marsh 0 stage (71.4%). Of the 16 cases of Marsh 2, 9 at the end of the diet completely normalized histology (56.2%) and 5 improved the picture reverting to Marsh 1 (31.2%). Therefore, the author concluded that, although the Marsh degrees 1 and 2 are not universally considered as CD, the improvement of symptoms, with or without improvement of histological lesions, may support the hypothesis that these patients are sensitive to gluten, thus justifying the opportunity of a dietary treatment.

## Conclusion

The spectrum of gluten-related disorders is wide, and encloses several conditions. For its singular pathogenesis, SNCD may be considered as an “immature” CD, where the global expression of autoantibodies is lacking. In a multi-step classification of gluten-related conditions (32), it has been proposed to place CD one step under the evident CD, due to a weaker involvement of adaptive immunity and a minor activation of tTG, as displayed in figure 2.

SNCD represents one of the most elusive conditions, due to the difficulties that a clinician may encounter to achieve a clear diagnosis. As above reported, SNCD is characterized by mucosal deposits of tTG and autoantibodies, and this finding could represent a diagnostic tool for a clear-cut definition of SNCD in the clinical practice. A quantitative assay of intestinal mRNA codifying for tTG has proven to be useful in the detection of SNCD and in the evaluation of the response to GFD in other and our experience (25, 33, 34). In the future, the standardization of this investigation could spread in clinical practice.

Finally, GFD in SNCD remains an open question. Several authors suggest a follow up with a gluten-containing diet, since a positivity of anti-tTG may successively occur (35). Additionally, patients often report disabling symptoms under gluten-containing diet, and this may prompt to propose a GFD in this defined subset of patients, i.e. the one with important gluten-related symptoms and positivity of HLA DQ2/8 (28). The choice of GFD administration is linked to the possibility of diagnosing SNCD with certainty, without advising it to large number of patients who may suffer from gluten sensitivity or irritable bowel syndrome, since exaggerate spreading of GFD may be deleterious for economic and clinical reasons. On these bases, a test helping the diagnosis of SNCD is justifiable and desirable. This issue, as well as other unsolved questions, make the universe of SNCD intriguing and exciting, but the few available studies do not state absolute key points in the diagnosis and treatment of SNCD. Therefore, further observations are needed in order to help the clinician in the decision-making process about this condition (36).

## References

[1] Fasano A, Catassi C (2012). Clinical practice. Celiac disease. N Engl J Med.

[2] Villanacci V, Ceppa P, Tavani E, Vindigni C, Volta U (2011). Coeliac disease: the histology report. Dig Liver Dis.

[3] Marsh MN (1992). Gluten, major histocompatibility complex, and the small intestine A molecular and immunobiologic approach to the spectrum of gluten sensitivity ('celiac sprue'). Gastroenterology.

[4] Armstrong D, Don-Wauchope AC, Verdu EF (2011). Testing for gluten-related disorders in clinical practice: the role of serology in managing the spectrum of gluten sensitivity. Can J Gastroenterol.

[5] Rubio-Tapia A, Hill ID, Kelly CP, Calderwood AH, Murray JA (2013). ACG clinical guidelines: diagnosis and management of celiac disease. Am J Gastroenterol.

[6] Rostami-Nejad M, Villanacci V, Hogg-Kollars S, Volta U, Manenti S, Reza-Zali M (2013). Endoscopic and histological pitfalls in the diagnosis of celiac disease: a multicentre study assessing the current practice. Rev Esp Enferm Dig.

[7] Rostami Nejad M, Karkhane M, Marzban A, Nazemalhosseini Mojarad E, Rostami K (2012). Gluten related disorders. Gastroenterol Hepatol Bed Bench.

[8] Abrams JA, Diamond B, Rotterdam H, Green PH (2004). Seronegative celiac disease: increased prevalence with lesser degrees of villous atrophy. Dig Dis Sci.

[9] Sulkanen S, Halttunen T, Laurila K, Kolho KL, Korponay-Szabó IR, Sarnesto A (1998). Tissue transglutaminase autoantibody enzyme-linked immunosorbent assay in detecting celiac disease. Gastroenterology.

[10] Tursi A, Brandimarte G, Giorgetti GM (2003). Prevalence of antitissue transglutaminase antibodies in different degrees of intestinal damage in celiac disease. J Clin Gastroenterol.

[11] Tursi A, Brandimarte G (2003). The symptomatic and histologic response to a gluten-free diet in patients with borderline enteropathy. J Clin Gastroenterol.

[12] Dickey W, Hughes DF, McMillan SA (2000). Reliance on serum endomysial antibody testing underestimates the true prevalence of coeliac disease by one fifth. Scand J Gastroenterol.

[13] Tursi A, Brandimarte G, Giorgetti G, Gigliobianco A, Lombardi D, Gasbarrini G (2001). Low prevalence of antigliadin and anti-endomysium antibodies in subclinical/silent celiac disease. Am J Gastroenterol.

[14] Rostami K, Kerckhaert J, Tiemessen R, von Blomberg BM, Meijer JW, Mulder CJ (1999). Sensitivity of antiendomysium and antigliadin antibodies in untreated celiac disease: disappointing in clinical practice. Am J Gastroenterol.

[15] Kurppa K, Ashorn M, Iltanen S, Koskinen LL, Saavalainen P, Koskinen O (2010). Celiac disease without villous atrophy in children: a prospective study. J Pediatr.

[16] Salmi TT, Collin P, Korponay-Szabó IR, Laurila K, Partanen J, Huhtala H (2006). Endomysial antibody-negative coeliac disease: clinical characteristics and intestinal autoantibody deposits. Gut.

[17] Korponay-Szabó IR, Halttunen T, Szalai Z, Laurila K, Király R, Kovács JB (2004). In vivo targeting of intestinal and extraintestinal transglutaminase 2 by coeliac autoantibodies. Gut.

[18] Tosco A, Maglio M, Paparo F, Rapacciuolo L, Sannino A, Miele E (2008). Immunoglobulin A anti-tissue transglutaminase antibody deposits in the small intestinal mucosa of children with no villous atrophy. J Pediatr Gastroenterol Nutr.

[19] Koskinen O, Collin P, Lindfors K, Laurila K, Mäki M, Kaukinen K (2010). Usefulness of small-bowel mucosal transglutaminase-2 specific autoantibody deposits in the diagnosis and follow-up of celiac disease. J Clin Gastroenterol.

[20] Koskinen O, Collin P, Korponay-Szabo I, Salmi T, Iltanen S, Haimila K (2008). Gluten-dependent small bowel mucosal transglutaminase 2-specific IgA deposits in overt and mild enteropathy coeliac disease. J Pediatr Gastroenterol Nutr.

[21] Kaukinen K, Peräaho M, Collin P, Partanen J, Woolley N, Kaartinen T (2005). Small-bowel mucosal transglutaminase 2-specific IgA deposits in coeliac disease without villous atrophy: a prospective and randomized clinical study. Scand J Gastroenterol.

[22] Chow MA, Lebwohl B, Reilly NR, Green PH (2012). Immunoglobulin A deficiency in celiac disease. J Clin Gastroenterol.

[23] Licinio R, Principi M, Amoruso A, Piscitelli D, Ierardi E, Di Leo A (2013). Celiac disease and common variable immunodeficiency: a familial inheritance?. J Gastrointestin Liver Dis.

[24] Malamut G, Verkarre V, Suarez F, Viallard JF, Lascaux AS, Cosnes J (2010). The enteropathy associated with common variable immunodeficiency: the delineated frontiers with celiac disease. Am J Gastroenterol.

[25] Montenegro L, Piscitelli D, Giorgio F, Covelli C, Fiore MG, Losurdo G (2014). Reversal of IgM deficiency following a gluten-free diet in seronegative celiac disease. World J Gastroenterol.

[26] Agarwal S, Mayer L (2013). Diagnosis and treatment of gastrointestinal disorders in patients with primary immunodeficiency. Clin Gastroenterol Hepatol.

[27] Biagi F, Bianchi PI, Zilli A, Marchese A, Luinetti O, Lougaris V (2012). The significance of duodenal mucosal atrophy in patients with common variable immunodeficiency: a clinical and histopathologic study. Am J Clin Pathol.

[28] Leffler D, Vanga R, Mukherjee R (2013). Mild enteropathy celiac disease: a wolf in sheep's clothing?. Clin Gastroenterol Hepatol.

[29] Adhi M, Farooq A, Hamid SA, Hasan R, Mamji S, Baloch AA (2009). Sero-negative celiac disease with dermatitis herpetiformes: a case report. Cases J.

[30] Singh P, Chaturvedi MK, Rangan P, Bhat AS (2013). Patients of celiac disease with mild villous atrophy are clinically similar to those with moderate to severe atrophy. Indian J Gastroenterol.

[31] Zanini B, Caselani F, Magni A, Turini D, Ferraresi A, Lanzarotto F (2013). Celiac disease with mild enteropathy is not mild disease. Clin Gastroenterol Hepatol.

[32] Troncone R, Jabri B (2011). Coeliac disease and gluten sensitivity. J Intern Med.

[33] Tosco A, Auricchio R, Aitoro R, Ponticelli D, Primario M, Miele E (2014). Intestinal titres of anti-tissue transglutaminase 2 antibodies correlate positively with mucosal damage degree and inversely with gluten-free diet duration in coeliac disease. Clin Exp Immunol.

[34] Maglio M, Tosco A, Auricchio R, Paparo F, Colicchio B, Miele E (2011). Intestinal deposits of anti-tissue transglutaminase IgA in childhood celiac disease. Dig Liver Dis.

[35] Auricchio R, Tosco A, Piccolo E, Galatola M, Izzo V, Maglio M (2014). Potential celiac children: 9-year follow-up on a gluten-containing diet. Am J Gastroenterol.

[36] Kaukinen K, Mäki M (2014). Coeliac disease in 2013: new insights in dietary-gluten-induced autoimmunity. Nat Rev Gastroenterol Hepatol.

